# PHB2 alleviates retinal pigment epithelium cell fibrosis by suppressing the AGE–RAGE pathway

**DOI:** 10.1515/biol-2022-0985

**Published:** 2024-11-04

**Authors:** Feng Chen, Xiaoxiao Cai, Ying Yu

**Affiliations:** Guangzhou Women and Children’s Medical Center, Guangzhou Medical University, No. 9, Jinsui Road, Tianhe District, Guangzhou, Guangdong, 510623, China; Guangzhou Women and Children’s Medical Center, Guangzhou Medical University, Guangzhou, Guangdong, 510623, China

**Keywords:** PHB2, TGF-β2, fibrosis, AGE, RAGE pathway

## Abstract

Fibrosis is the primary cause of retinal detachment and visual decline. Here, we investigated the role of Prohibitin 2 (PHB2) in modulating fibrosis in ARPE-19 cells stimulated by transforming growth factor (TGF)-β2. The proliferation, migration, and apoptosis of ARPE-19 cells were evaluated using 3-(4,5-dimethylthiazol-2-yl)-2,5-diphenyltetrazolium bromide, wound healing, and flow cytometry assays, and levels of fibrosis-associated and pathway-related proteins were determined by performing western blotting. To examine the mechanisms underlying ARPE-19 cell fibrosis, we performed RNA sequencing, protein–protein interaction network, and enrichment analyses. We detected increases in the expression of the fibrosis-related proteins fibronectin and collagen I in response to TGF-β2 treatment, whereas the expression of PHB2 was downregulated. PHB2 overexpression suppressed the proliferation and migration of TGF-β2-stimulated ARPE-19 cells, promoted apoptosis, and inhibited fibrosis and Smad and non-Smad pathways. PHB2 overexpression inhibited the advanced glycation end-product (AGE)–receptor of advanced glycation end-product (RAGE) pathway activated by TGF-β2 treatment, which contributed to enhancing the effects of PHB2 on cellular processes, fibrosis, and Smad and non-Smad pathways. Conversely, exogenous application of AGE counteracted the effects of PHB2 overexpression. We conclude that by suppressing the AGE–RAGE pathway, PHB2 exerts an inhibitory effect on TGF-β2-induced fibrosis in ARPE-19 cells.

## 1 Introduction

The retinal pigment epithelium (RPE) is a single layer of pigment cells lying between the neuroretina and choroid that contributes to maintaining visual function [1]. Dedifferentiation, migration, and growth of RPE cells can eventually lead to the development of myofibroblasts, thereby inducing proliferative vitreoretinopathy [2]. Subretinal fibrosis inevitably leads to severe and irreversible visual impairment [3,4], and currently, patients with subretinal fibrosis seldom benefit from common anti-vascular endothelial growth factor therapy [5]. Consequently, there is a pressing need to identify effective treatments for this fibrosis.

Transforming growth factor-beta (TGF-β), which has been established to regulate immune responses and inflammatory processes, is considered one of the major factors contributing to the development of fibrosis [6,7], and Smad- and non-Smad-dependent pathways have been identified as the major transmitters of TGF-β signaling [8]. These pathways contribute to the regulation of cell proliferation, thereby playing an intrinsic basal role in the fibrotic response and development of RPE cells [9,10]. The Smad-dependent pathways mainly involve Smad2 and Smad3 that form trimers with Smad4 and undergo nuclear translocation to regulate the expression of related genes, whereas non-Smad-dependent pathways include the Akt, PI3K, and MAPK (e.g., JNK, p38MAPK, and ERK1/2) signaling pathways [11,12]. However, the specific mechanisms whereby TGF-β induces fibrosis in RPE cells have yet to be sufficiently determined.

Prohibitin (PHB) 1 and PHB2 are two PHB subunits found mainly within the inner mitochondrial membrane, nucleus, cytoplasm, plasma membrane, endoplasmic reticulum, and macrophage phagosomes [13,14], wherein they play roles in the regulation of aging and the development of proliferative, degenerative, and metabolic diseases [15,16,17,18]. PHB2 has been identified as an autophagy receptor expressed on the inner mitochondrial membrane that induces mitochondrial autophagy following the rupture of the outer mitochondrial membrane [19]. In diabetic nephropathy, mitochondrial autophagy in tubular epithelial cells of the kidney contributes to reductions in damaged mitochondria and interstitial fibrosis [20]. Additionally, the overexpression of PHB has been demonstrated to inhibit apoptosis and the production of reactive oxygen species, thereby suppressing renal tubule atrophy and fibrosis following renal transplantation [21]. Furthermore, inhibition of mitochondrial autophagy has been found to ameliorate myocardial fibrosis in rats with myocardial infarction, whereas downregulation of PHB2 has been shown to inhibit the fibrosis of cardiac fibroblasts [22,23]. To date, however, the role of PHB2 in subretinal fibrosis has yet to be determined.

Advanced glycation end-products (AGEs) are heterogeneous toxic compounds produced when proteins are exposed to reducing sugars [24]. Endogenous formation of AGEs can promote oxidative stress and protein modification, thereby contributing to the regulation of inflammatory gene expression and the subsequent enhanced production of inflammatory cytokines [25]. Additionally, the interaction between AGEs and their respective receptors (RAGEs) has been demonstrated to promote the dysregulation of cell signaling and inflammatory responses [26]. Moreover, it has been established that the AGE–RAGE pathway plays a prominent role in the development of fibrosis, including that of cardiac, renal, hepatic, and pulmonary tissues [27,28,29], and it has also been reported that AGEs are pathological factors involved in the occurrence and development of diabetic retinopathy and have accordingly been identified as therapeutic targets for blocking the progression of this disease [30]. However, although AGE synthesis has been shown to promote biochemical impairment in retinal tissues, mediated by both RAGE-dependent and -independent receptors [31,32], the role of the AGE–RAGE pathway in subretinal fibrosis has yet to be established.

In this study, we sought to determine the potential regulatory mechanisms underlying the development of subretinal fibrosis and thereby provide a theoretical basis and therapeutic targets for the treatment of this disease. To this end, we used TGF-β to induce fibrosis in RPE cells, and by overexpressing PHB2, we investigated the role of this protein in RPE cell fibrosis. In addition, to elucidate the mechanisms whereby PHB2 contributes to the development of fibrosis, we performed RNA sequencing.

## 2 Methods

### 2.1 Cell culture and transfection

Human RPE cells (ARPE-19: iCell-h020; iCell Bioscience Inc, Shanghai, China) were incubated in DMEM/F12 medium (Thermo Fisher Scientific, Waltham, MA, USA) supplemented with 10% fetal bovine serum (Gibco, Grand Island) and 1% Penicillin/Streptomycin Solution (Invitrogen, Carlsbad, CA, USA) in a 5% CO_2_ incubator at 37°C. After reaching 80% confluence, the cells were incubated with 10 ng/mL recombinant human TGF-β2 (R&D Systems, Minneapolis, MN, USA). PHB2-overexpressing lentiviral and control vectors were constructed, and Lipofectamine 3000 Reagent (Thermo Fisher Scientific) was used for cell transfection.

### 2.2 RNA-sequencing

RNA was extracted from ARPE-19 cells using a MasterPure Complete DNA and RNA Purification Kit (MC85200; Epicenter, Madison, WI, USA), and the RNA thus obtained was analyzed using an Agilent 2100 Bioanalyzer (Santa Clara, California, USA). RNA library construction was performed using an Illumina Tru Seq RNA library construction kit to convert RNA to cDNA and subsequently attach sequencing adapters. The RNA was sequenced using the Illumina HiSeq X Ten sequencing platform.

### 2.3 Analysis of differentially expressed genes

The criteria for defining differential gene expression were as follows: differential expression multiple |log2FoldChange| > 1 and significant *P*-value <0.05. Two-way clustering analysis was performed using the pheatmap software package in R for the identified differential expressed genes (DEGs). Gene Ontology (GO) and Kyoto Encyclopedia of Genes and Genomes (KEGG) enrichment analyses were performed using DAVID (https://david.ncifcrf.gov/).

### 2.4 Cell treatment

Induction of fibrosis using TGF-β2 was performed as described in Section 2.1. To assess the effects of the AGE–RAGE pathway on fibrosis in RGE cells, we added 20 μg/mL of the AGE inhibitor ALT-711 (MedChemExpress, New Jersey, USA) to cell culture medium containing isopycnic DMSO and maintained for 24 h. AGE-modified bovine serum albumin was prepared by incubation with bovine serum albumin and d-glucose for 8 weeks and was subsequently purified, as previously described [33], and to investigate the regulatory effects of PHB2 on the AGE–RAGE pathway, cells were treated with 100 μg/mL AGE.

### 2.5 Determination of cell proliferation

To assess cell proliferation, we performed the 3-(4,5-dimethylthiazol-2-yl)-2,5-diphenyltetrazolium Bromide (MTT) assay. ARPE-19 cells were initially seeded in the wells of 96-well plates (1 × 10^4^/well) and incubated in a 5% CO_2_ incubator at 37℃ for 24 h. Subsequently, 20 μL of 0.5 mg/mL MTT reagent (Solarbio, Beijing, Shanghai) was added to the cells followed by incubation at 37°C for 2 h. After subsequently removing the MTT reagent, DMSO (150 mL) was also added and the cells were incubated for 10 min, after which the absorbance at 490 nm was recorded using a microplate reader.

### 2.6 Wound healing assay

The ARPE-19 cells were seeded into the six-well plates (5 × 10^5^/well) and cultured until reaching 80% confluence, and which point, a “wound” was created in the cell layer using a pipette tip. After washing with PBS, the cells were then seeded in a serum-free medium and incubated at 37°C for 24 h. The wound was subsequently observed under a microscope at 0 and 24 h, and cell migration capacity was analyzed using ImageJ software.

### 2.7 Apoptosis assessment

Apoptosis was evaluated by flow cytometry using an Annexin V-FITC Apoptosis Assay Kit (Vazyme, Nanjing, China). ARPE-19 cells were resuspended in 1× binding buffer and mixed with Annexin V-FITC and PI staining solutions. Fluorescence intensity was measured by flow cytometry and analyzed using FlowJo software.

### 2.8 Western blotting

Proteins were extracted from ARPE-19 cells using RIPA lysis buffer (Biosharp, Beijing, China), the concentrations of which were determined using a Pierce BCA Protein Assay Kit (Thermo Fisher Scientific). The isolated proteins were separated on 10% sodium dodecyl sulfate-polyacrylamide gels and transferred onto polyvinylidene fluoride membranes. Having initially blocked the membranes with 5% skim milk, they were incubated overnight at 4°C with the following selected primary antibodies: anti-Fibronectin (1:2,000; Abcam, Cambridge, MA, USA), anti-collagen I (1:2,000; Abcam), anti-PHB2 (1:2,000; Abcam), anti-Smad (1:2,000; Abcam), anti-E2F11 (1:2,000; Abcam), anti-PI3K (1:2,000; Abcam), anti-AKT (1:2,000; Abcam), anti-MEK1/2 (1:2,000; Abcam), anti-AGE (1:2,000; Abcam), anti-RAGE (1:2,000; Abcam), and anti-β-actin (1:1,000, Abcam). In the following day, the membranes were incubated with anti-rabbit IgG secondary antibody (1:5,000; Abcam) for 1 h at room temperature. Bands were scanned using a Tanon 5200 automatic chemiluminescence image analysis system (Shanghai, China), with ECL luminescent solution being used for color development.

### 2.9 Statistical analysis

All data, expressed as the mean ± standard deviation, were statistically analyzed using GraphPad Prism 7.0. Comparisons between two groups were analyzed using *t*-tests and a one-way analysis of variance was used for the analysis of multi-group comparisons, followed by Tukey’s multiple comparison test. Statistical significance was set at *P* < 0.05.

## 3 Results

### 3.1 TGF-β2 induces fibrosis and PHB2 downregulation in ARPE-19 cells

**Figure 1:**
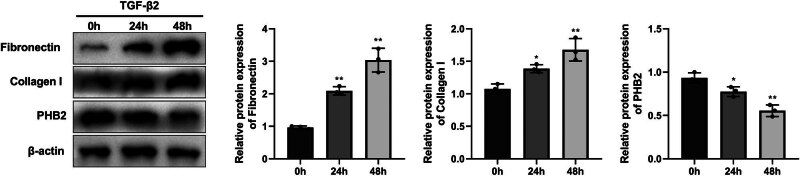
PHB2 was downregulated in TGF-β2-induced ARPE-19 cells. Western blotting detected the expressions of fibronectin, collagen I, and PHB2 in TGF-β2-induced ARPE-19 cells. *P < 0.05 compared with 0 h. **P < 0.01 compared with 0 h.

Following treatment with TGF-β2 for 24 and 48 h, we detected significantly higher levels of fibronectin and collagen I expression, whereas significant reduction was detected in the levels of PHB2 (Figure 1).

### 3.2 PHB2 overexpression inhibits proliferation and migration and promotes apoptosis of TGF-β2-induced ARPE-19 cells

**Figure 2:**
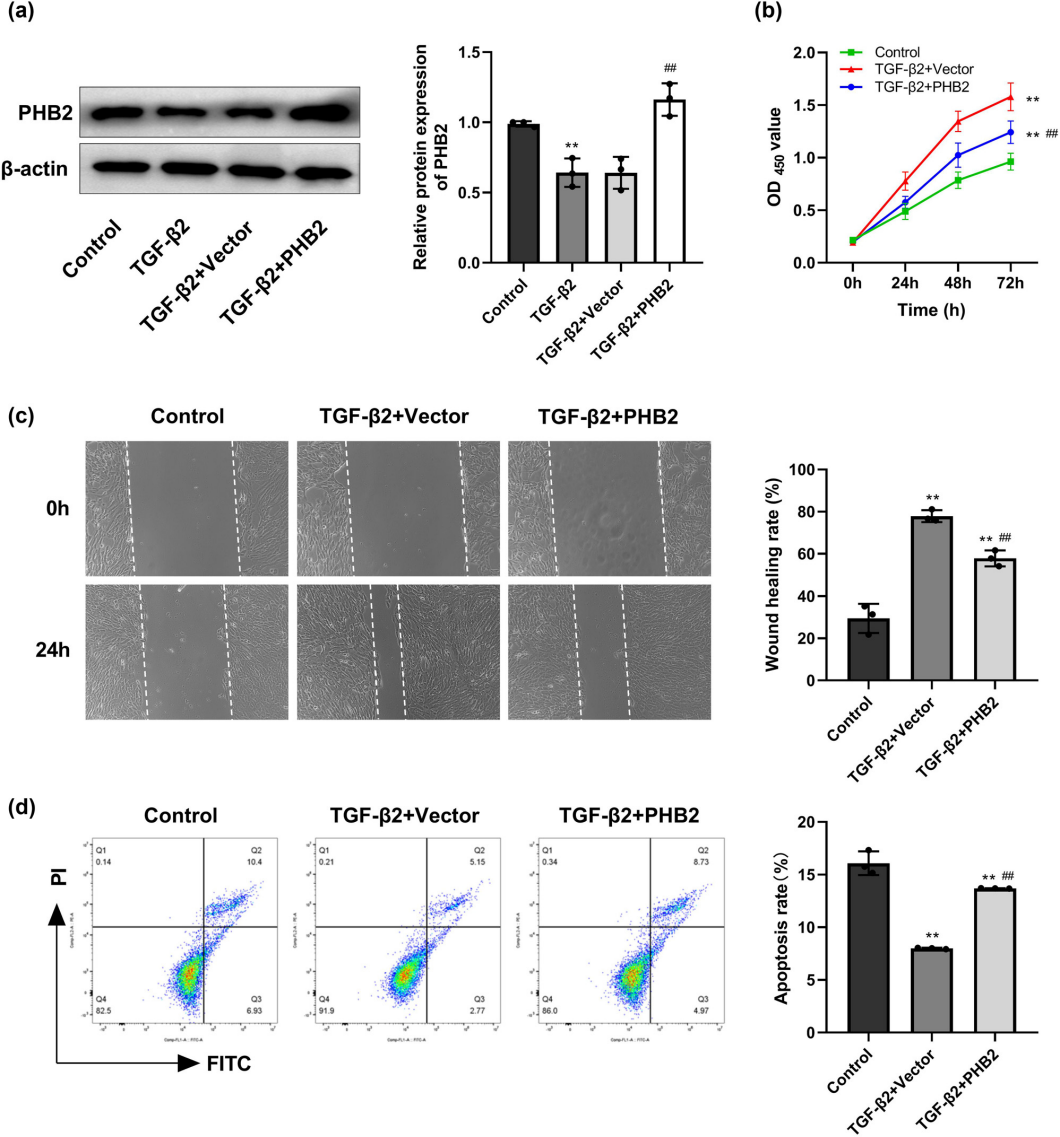
Overexpression of PHB2 inhibited cell proliferation, migration, and promoted cell apoptosis of TGF-β2-induced ARPE-19 cells. (a) Western blotting detected the expression of PHB2. (b) Cell proliferation was detected by MTT assay. (c) Cell migration was detected by wound-healing assay. (d) Flow cytometry was used to detect apoptosis. **P < 0.01 compared with the control group. ^##^ *P* < 0.01 compared with the TGF-β2 + vector group.

The transfection efficiency was verified by Western blotting, which indicated that PHB2 expression in the TGF-β2 group was considerably lower than that in the control group. Whereas we detected no substantial difference between the TGF-β2 + vector and TGF-β2 groups with respect to PHB2 expression, significantly higher levels of PHB2 expression were observed in the TGF-β2 + PHB2 group (Figure 2a).

Compared with the control group, a significant promotion of ARPE-19 cell proliferation and migration was detected in the TGF-β2 + vector and TGF-β2 + PHB2 groups. Conversely, compared with the TGF-β2 + vector group, cell proliferation and migration were found to be significantly inhibited in the TGF-β2 + PHB2 group (Figure 2b and c). The results of flow cytometry revealed notably reduced rates of apoptosis in the TGF-β2 + vector and TGF-β2 + PHB2 groups compared with the control group, whereas the levels of apoptosis in the TGF-β2 + PHB2 group were considerably higher than those in the TGF-β2 + vector group (Figure 2d).

### 3.3 PHB2 overexpression inhibits Smad- and non-Smad-dependent pathways

**Figure 3:**
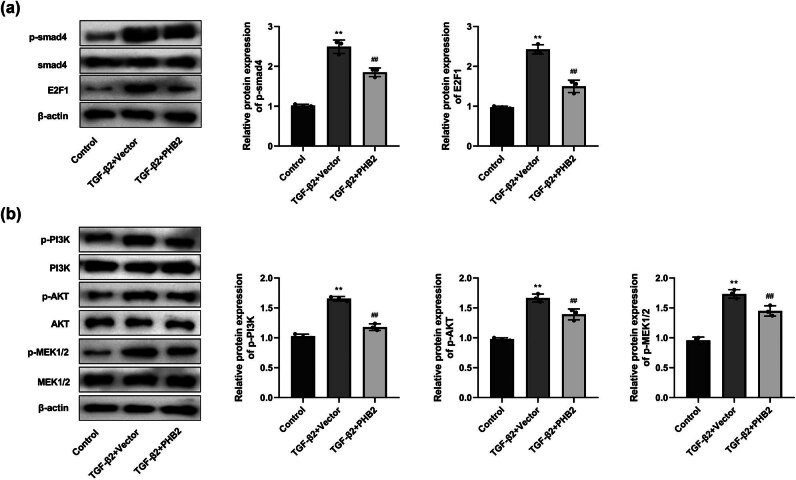
Overexpression of PHB2 regulated Smad-dependent and non-Smad-dependent pathways. (a) Western blotting detected the expressions of Smad 4 and E2F1. (b) Western blotting detected the expressions of PI3K, AKT, and MEK1/2. **P < 0.01 compared with the control group. ^##^ *P* < 0.01 compared with the TGF-β2 + vector group.

Given that Smad- and non-Smad-dependent pathways have been established to mediate TGF-β signal transmission, we examined the expression of phosphorylated Smad (p-Smad4 and E2F1) and non-Smad (p-PI3K, p-AKT, and p-MEK1/2)-dependent pathway proteins. Compared with the control group, we accordingly detected significantly higher levels of p-Smad4 and E2F1 expression in the TGF-β2 + vector group, whereas compared with the TGF-β2 + vector group, there were significant reductions in the expression of p-Smad4 and E2F1 in the TGF-β2 + PHB2 group (Figure 3a). Moreover, compared with the control group, significantly higher levels of p-PI3K, p-AKT, and p-MEK1/2 were detected in the TGF-β2 + vector group. However, compared with the TGF-β2 + vector group, there were significant reductions in the expression of p-PI3K, p-AKT, and p-MEK1/2 in the TGF-β2 + PHB2 group (Figure 3b).

### 3.4 DEG selection

A total of 2387 DEGs were selected from the TGF-β2 + vector and TGF-β2 + PHB2 groups, among which 2,164 and 223 genes were significantly up- and downregulated, respectively (Figure S1a). The cluster map presented in Figure S1b showed the bidirectional clustering of both groups of DEGs.

### 3.5 GO and KEGG enrichment analyses

GO functional analysis revealed that in the cell component category, terms associated with “organelles” and “nuclei” were significantly enriched with DEGs, whereas in the biological processes category, the terms “nucleic acid metabolism” and “heterocyclic metabolism” were significantly enriched. Furthermore, “nucleic acid binding” and “heterocyclic compound binding” were identified as significantly enriched terms in the molecular functions category (Figure S2a). In addition, KEGG pathway analysis revealed that DEGs were mainly concentrated in the TGF-β, AGE–RAGE, TNF, P53, Il-17, and PI3K–Akt signaling pathways (Figure S2b).

### 3.6 TGF-β2 activates the AGE–RAGE pathway in ARPE-19 cells

**Figure 4:**
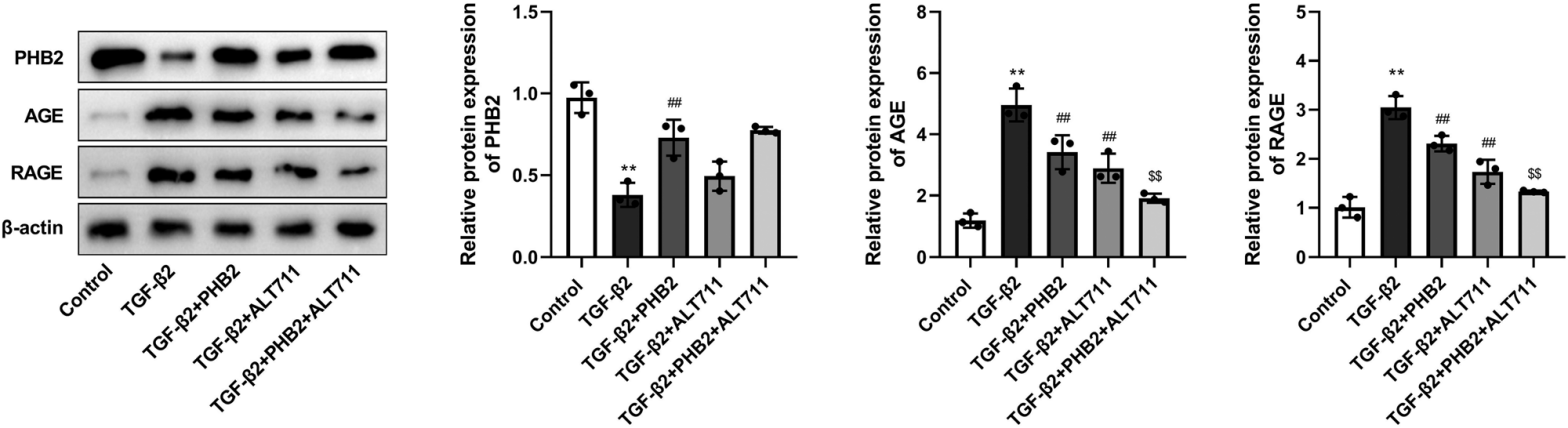
TGF-β2 induced AGE–RAGE pathway activation in ARPE-19 cells. Western blotting detected the expressions of PHB2, AGE, and RAGE. **P < 0.01 compared with the control group. ^##^ *P* < 0.01 compared with the TGF-β2 group. ^$$^ *P* < 0.01 compared with the TGF-β2 + PHB2 group.

As a target pathway in the present study, we selected the AGE–RAGE pathway. Compared with the control group, the expression of PHB2 in the TGF-β2 group was significantly lower, whereas the levels of AGE and RAGE were considerably higher. Compared with the TGF-β2 group, we detected a significant increase in the expression of PHB2 in the TGF-β2 + PHB2 group, whereas there were significant reductions in the levels of AGE and RAGE. However, we detected no significant difference between the TGF-β2 + ALT711 and TGF-β2 groups with respect to the expression of PHB2, whereas the levels of AGE and RAGE were significantly reduced in the former. Furthermore, there were no significant differences between the TGF-β2 + PHB2 + ALT711 and TGF-β2 + PHB2 groups regarding the expression of PHB2, whereas the levels of AGE and RAGE were significantly lower in the former (Figure 4).

### 3.7 PHB2 inhibits fibrosis via suppression of the AGE–RAGE pathway

**Figure 5:**
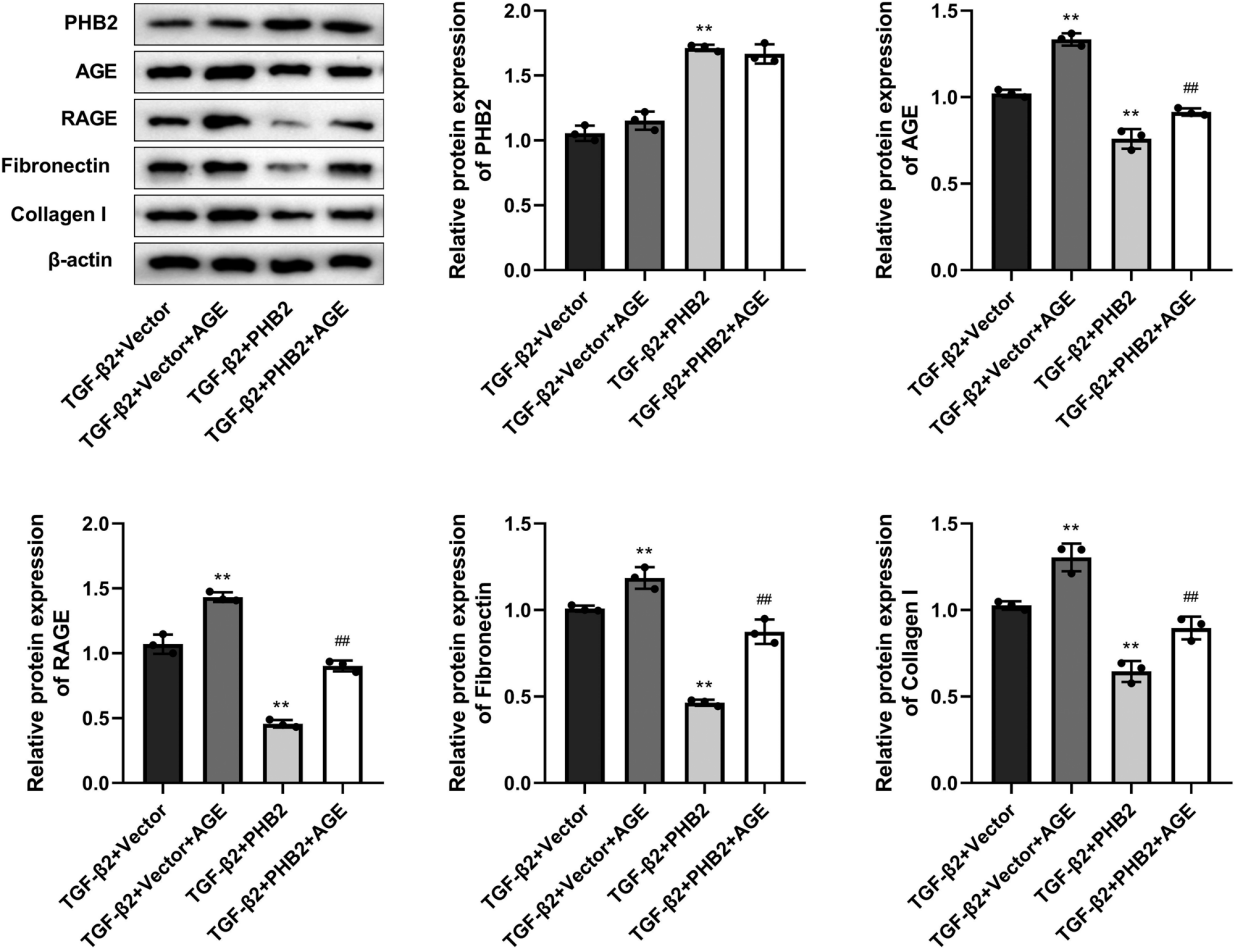
PHB2 inhibited the expression of fibrosis-related proteins fibronectin and collagen I via the AGE–RAGE signaling pathway. Western blotting detected the expressions of PHB2, AGE, RAGE, fibronectin, and collagen I. **P < 0.01 compared with the TGF-β2 + vector group. ^##^ *P* < 0.01 compared with the TGF-β2 + PHB2 group.

Compared with those in the TGF-β2 + vector group, we detected considerably lower levels of AGE, RAGE, collagen I, and fibronectin expression in the TGF-β2 + PHB2 group. In contrast, the expression of AGE, RAGE, and fibrosis-related proteins (fibronectin and collagen I) increased significantly in response to AGE treatment (Figure 5).

### 3.8 PHB2 inhibits the proliferation and migration of ARPE-19 cells but promotes apoptosis by suppressing the AGE–RAGE pathway

**Figure 6:**
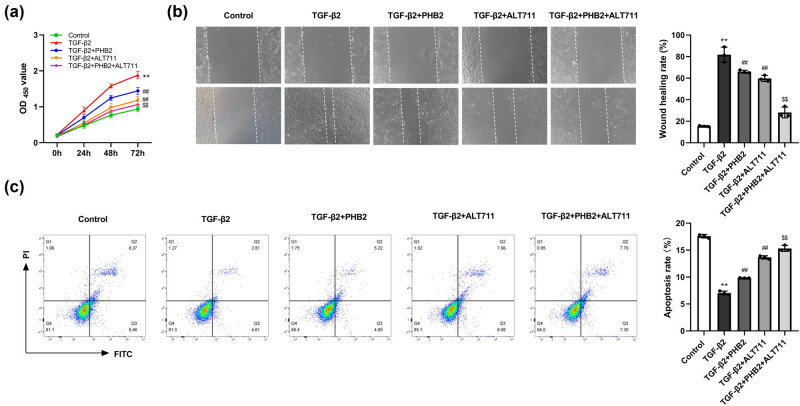
AGE–RAGE pathway promoted the proliferation and migration and inhibited cell apoptosis of TGF-β2-induced ARPE-19 cells. (a) MTT assay detected cell proliferation. (b) Cell migration was detected by wound-healing assay; (c) Apoptosis was detected by flow cytometry. **P < 0.01 compared with the control group. ^##^ *P* < 0.01 compared with the TGF-β2 group. ^$$^ *P* < 0.01 compared with the TGF-β2 + PHB2 group.

Compared to the control group, we observed that the proliferation and migration of ARPE-19 cells were apparently promoted in the TGF-β2 group. However, compared with the TGF-β2 group, cell proliferation and migration in the TGF-β2 + PHB2 and TGF-β2 + ALT711 groups were significantly inhibited. Furthermore, compared with the TGF-β2 + PHB2 group, we detected a significant suppression of cell proliferation and migration in the TGF-β2 + PHB2 + ALT711 group (Figure 6a and b). Flow cytometry revealed that compared with that in the control group, there was a significant reduction in the rate of cellular apoptosis in the TGF-β2 group. However, compared with the TGF-β2 group, we detected significantly higher rates of apoptosis in the TGF-β2 + PHB2 and TGF-β2 + ALT711 groups, and compared with that in the TGF-β2 + PHB2 group, significantly higher rates of apoptosis were detected in the TGF-β2 + PHB2 + ALT711 group (Figure 6c).

**Figure 7:**
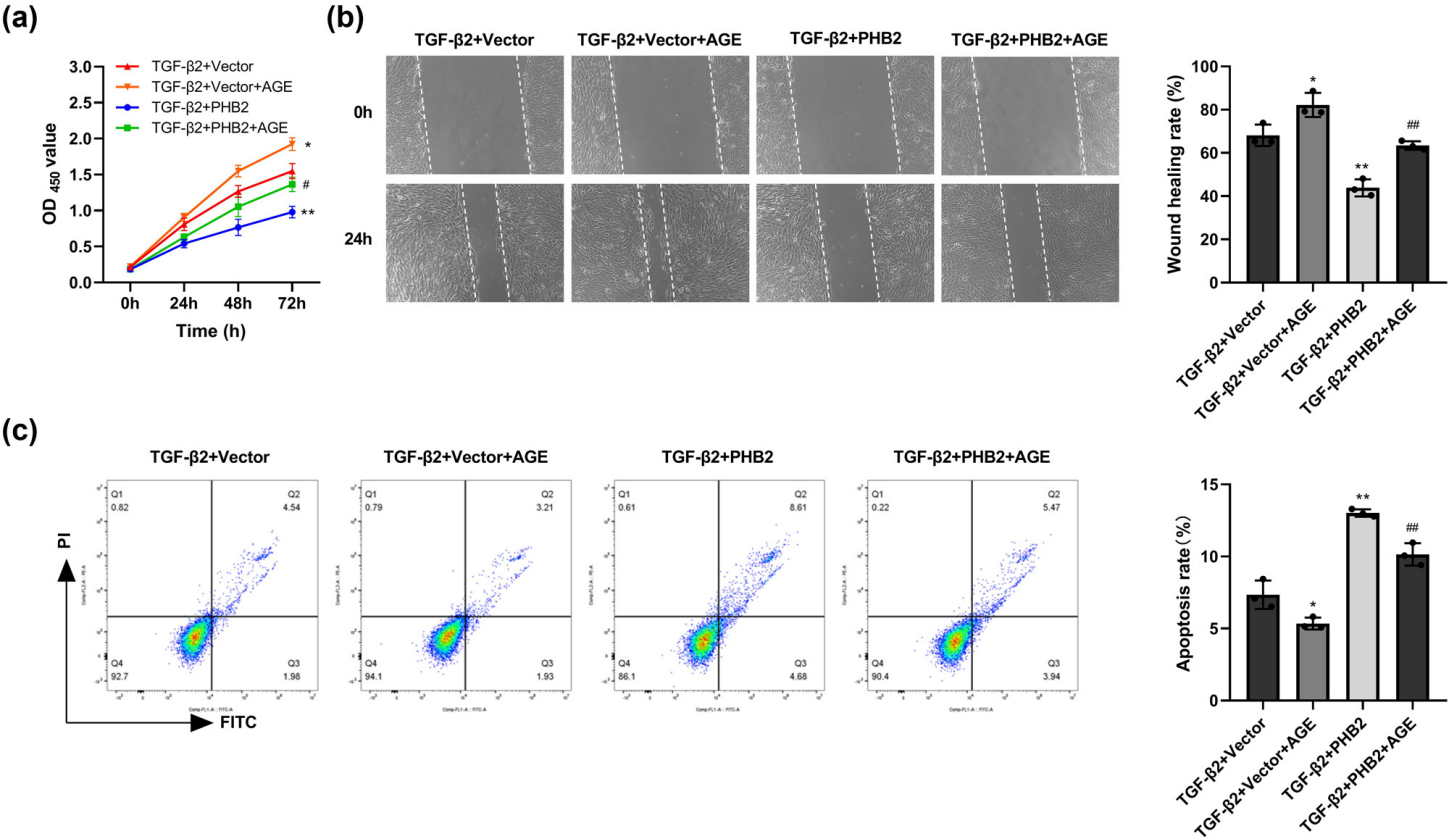
PHB2 inhibited proliferation, migration, and promoted apoptosis through the AGE–RAGE pathway of ARPE-19 cells. (a) MTT assay detected cell proliferation. (b) Cell migration was detected by wound-healing assay. (c) Apoptosis was detected by flow cytometry. **P < 0.01 compared with the TGF-β2 + vector group. ^##^ *P* < 0.01 compared with the TGF-β2 + PHB2 group.

Compared to the TGF-β2 + Vector group, cell proliferation and migration were significantly reduced in the TGF-β2 + PHB2 group. After treatment with AGE, cell proliferation and migration in the TGF-β2 + PHB2+AGE group increased, compared to the TGF-β2 + PHB2 group. Compared to the TGF-β2 + Vector group, cell apotosis were significantly promoted in the TGF-β2 + PHB2 group. After treatment with AGE, cell apoptosis in the TGF-β2 + PHB2+AGE group was significantly inhibited, compared to the TGF-β2 + PHB2 group. (Figure 7c).

### 3.9 PHB2 inhibits Smad- and non-Smad-dependent pathways by suppressing AGE–RAGE pathways

**Figure 8:**
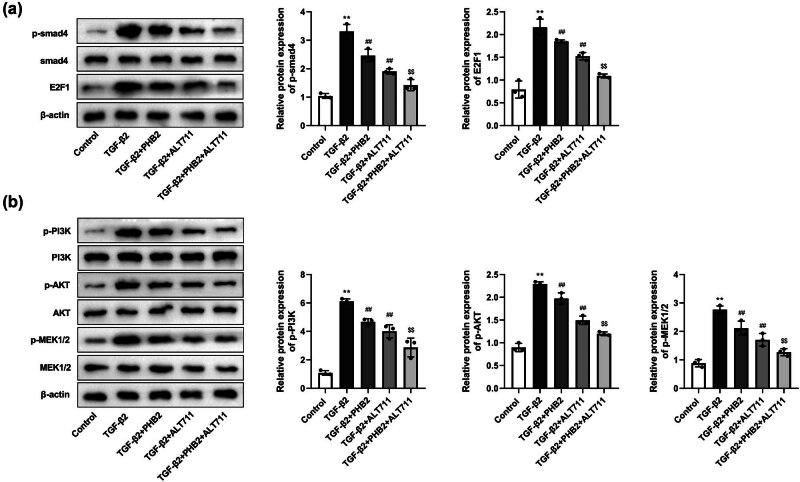
AGE–RAGE pathway regulated the expression of proteins in Smad-dependent and non-Smad-dependent pathway proteins. (a) Western blotting detected the expressions of Smad4 and E2F1. (b) Western blotting detected the expressions of PI3K, AKT, and MEK1/2. **P < 0.01 compared with the control group. ^##^ *P* < 0.01 compared with the TGF-β2 group. ^$$^ *P* < 0.01 compared with the TGF-β2 + PHB2 group.

Compared with those in the control group, we detected apparently higher levels of p-Smad4 and E2F1 in the TGF-β2 group, whereas compared with the TGF-β2 group, there were significant reductions in the expression of p-Smad 4 and E2F1 in the TGF-β2 + PHB2 and TGF-β2 + ALT711 groups. Moreover, the levels of p-Smad4 and E2F1 expression in the TGF-β2 + PHB2 + ALT711 group were found to be markedly lower than those in the TGF-β2 + PHB2 group (Figure 8a). With respect to the non-Smad-dependent pathways, we detected notable increases in the expression of p-PI3K, p-AKT, and p-MEK1/2 in the TGF-β2 group compared with those in the control group, whereas compared with the TGF-β2 group, significantly lower levels of p-PI3K, p-AKT, and p-MEK1/2 expression were detected in the TGF-β2 + PHB2 and TGF-β2 + AL T711 groups. Furthermore, in the TGF-β2 + PHB2 + AL T711 group, the levels of p-PI3K, p-AKT, and p-MEK1/2 expression were found to be significantly lower than those in the TGF-β2 + PHB2 group (Figure 8b).

**Figure 9:**
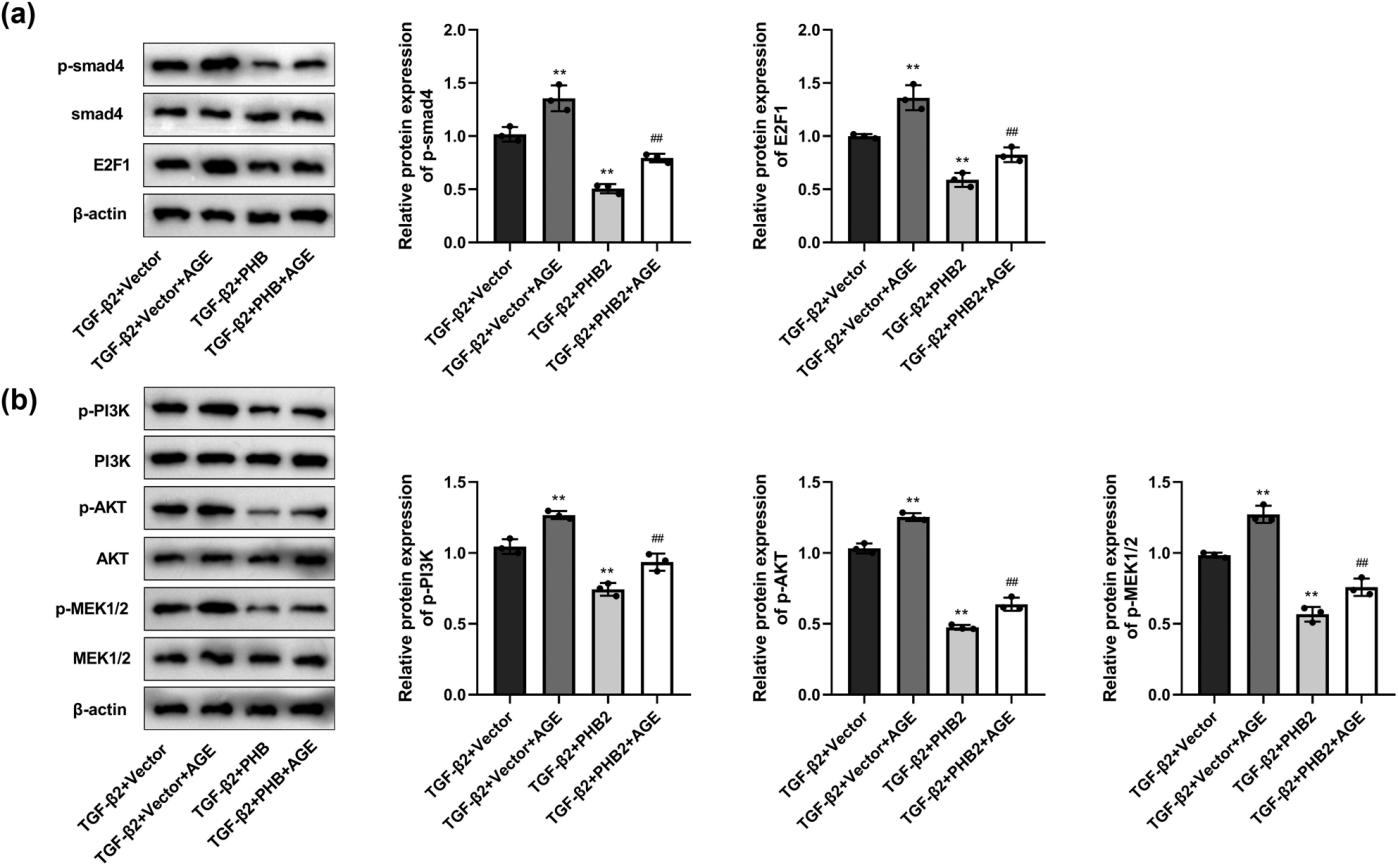
PHB2 regulated the expression of proteins in Smad-dependent and non-Smad-dependent pathway proteins through the AGE–RAGE pathway. (a) Western blotting detected the expressions of Smad4 and E2F1. (b) Western blotting detected the expressions of PI3K, AKT, and MEK1/2. **P < 0.01 compared with the TGF-β2 + vector group. ^##^ *P* < 0.01 compared with the TGF-β2 + PHB2 group.

We also established that the levels of p-Smad4 and E2F1 expression in the TGF-β2+PHB2 group were significantly lower than those in the TGF-β2+Vector group. However, we detected an increase in the expression of p-Smad4 and E2F1 in response to AGE treatment in TGF-β2+PHB2+AGE group, compared to the TGF-β2+PHB2 group (Fig. 9A). Additionally, compared with those in the TGF-β2+Vector group, we detected significant reductions in the levels of p-PI3K, p-AKT, and p-MEK1/2 expression in the TGF-β2+PHB2 group. Similar to the aforementioned Smad proteins, AGE treatment was observed to promote the expression of p-PI3K, p-AKT, and p-MEK1/2 in TGF-β2+PHB2+AGE group, compared to the TGF-β2+PHB2 group (Fig. 9B).

## 4 Discussion

Subretinal fibrosis, a major pathological feature of neovascular age-related macular degeneration, can lead to structural and functional impairment of RPE cells and photoreceptors, thereby causing an irreversible loss of central vision [8]. In this study, we observed fibrosis and a reduction in PHB2 expression in TGF-β2-induced ARPE-19 cells, and on the basis of transcriptome sequencing, we identified the AGE–RAGE pathway as a potential mechanism whereby PHB2 inhibits subretinal fibrosis. Furthermore, overexpression of PHB2 was found to suppress fibrosis in ARPE-19 cells by inhibiting the AGE–RAGE pathway, which was associated with a disruption of ARPE-19 cell proliferation and migration, along with increased levels of apoptosis. In addition, by suppressing the AGE–RAGE pathway, the overexpression of PHB2 was found to contribute to the downregulated expression of both Smad (Smad4 and E2F1) and non-Smad (PI3K, AKT, and MEK1/2)-dependent pathway proteins.

PHB2 has been established to be a key mitochondrial receptor involved in targeted mitochondrial autophagic degradation [19]. It is noteworthy that mitochondrial dysfunction is increasingly being found to be associated with common age-related ophthalmic diseases, including diabetic retinopathy, age-related macular degeneration, and glaucoma [34]. In streptozotocin-treated mouse models and tissues from patients with diabetes, PHB expression has been found to be downregulated and thereby serves as a biomarker for diabetic retinopathy [35]. Additionally, the loss of PHB2 impairs the stability of Optic Atrophy 1 (OPA1), and mutations in OPA1 have been shown to be associated with dominant optic atrophy, characterized by a gradual loss of retinal ganglion cells [36]. In the present study, we observed that TGF-β2 treatment led to a reduction in PHB2 expression and induced fibrosis in ARPE-19 cells, whereas the overexpression of PHB2 was found to inhibit cell proliferation and migration, although also had the effect of enhancing apoptosis. The role of PHB2 in cellular and organ fibrosis has been identified in previous studies. For example, rats with renal interstitial fibrosis were found to be characterized by elevated levels of profibrotic components and reductions in the expression of PHB2, which was inversely correlated with the extent of fibrosis [37]. PHB2 has been shown to ameliorate DOX-induced cardiomyopathy by inhibiting interstitial fibrosis and restoring the mitochondrial complex I function by interacting with NDUFV2 [38]. Collectively, the findings of the present and previous studies provide convincing evidence to indicate that PHB2 plays a pivotal role in both retinal pathologies and organ fibrosis, and by inhibiting fibrosis, the overexpression of PHB2 can contribute to alleviating retinopathy-induced retinal fibrosis.

TGF-β is widely considered to function as a master regulator of tissue fibrosis [39]. In the present study, we found that overexpression of PHB2 inhibited Smad (Smad4/E2F1) and non-Smad (PI3K/AKT/MEK1/2)-dependent pathways in ARPE-19 cells that had been induced by TGF-β1. In TGF-β1-induced cell models, it has previously been established that the abnormal expression of TGF-β1 and phosphorylation of Smad2 and Smad3 are downregulated, thereby tending to indicate that inhibition of the TGF-β/Smad pathway prevents epithelial–mesenchymal transition in fibrosis [40]. In this regard, it has been demonstrated that TGF-β2 promotes subretinal fibrosis by inducing the transformation of pericytes to myofibroblasts via the Smad2/3 and Akt/mTOR pathways [41]. Additionally, specific deletion of Smad4 in hepatocytes has been found to reduce liver inflammation and fibrosis, reverse the suppression of epithelial–mesenchymal transformation, and inhibit hepatocyte proliferation and migration [42]. Non-Smad-dependent pathways have similarly been established to be associated with the development of fibrosis. For example, activation of the PI3K/AKT/ERK signaling pathway has been detected in the retinal tissue of myopic guinea pigs, thereby exacerbating fibrotic lesions and reducing retinal thickness, ultimately resulting in physiological malfunction of the retina [43]. Similarly, activation of the MEK1/2-ERK1/2 signaling pathway has been shown to promote extracellular matrix deposition, oxidative stress damage, and cardiac fibrosis [44]. Studies conducted to date have also established that PHB2 expression is negatively correlated with the degree of renal interstitial fibrosis and levels of TGF-β1 in the tissues of fibrotic rats. Collectively, these findings provide evidence to indicate the pivotal role of PHB2 in the TGF-β-induced fibrotic process [37]. On the basis of our findings in the present study, we conclude that the overexpression of PHB2 contributes to inhibiting Smad- and non-Smad-dependent pathways in TGF-β2-induced ARPE-19 cells, thereby inhibiting fibrosis.

In this study, we identified the AGE–RAGE pathway, which was activated in TGF-β2-induced ARPE-19 cells, as the pathway associated with the fibrosis-related regulatory activity of PHB2, The AGE–RAGE pathway has been established to be a key regulatory pathway in retinal diseases. For example, network pharmacology analysis has revealed that the anti-diabetic retinopathy effect of astragalus in diabetic complications is mainly mediated through the AGE–RAGE signaling pathway [45]. Furthermore, this pathway is considered to be a potentially key target for the treatment of diabetic retinopathy [46]. Moreover, the role of the AGE–RAGE pathway in fibrosis of a range of different organs and tissues has been reported. For example, DEGs in fibrotic breast tissues have been found to be significantly enriched in the AGE–RAGE pathway, thereby indicating that this pathway may play a key role in the development of fibrosis [47]. Consistent with this assumption, oral administration of pomegranate fruit extract has been demonstrated to reduce necrotizing inflammation of the portal vein and suppress fibrosis by inhibiting the expression of AGEs and RAGEs [28]. Similarly, by modulating the AGE–RAGE/HMGB-1 signaling pathway, which affects oxidative stress, inflammation, and fibrosis, artemisinin has been shown to contribute to the amelioration of diabetic cardiomyopathy [48]. Notably, in the present study, we found that by suppressing the AGE–RAGE pathway, the overexpression of.PHB2 in TGF-β2-stimulated ARPE-19 cells inhibited fibrosis, as well as inhibiting cell proliferation and migration and promoting apoptosis. Similarly, by inhibiting the AGE–RAGE pathway, PHB2 also blocked both Smad- and non-Smad-dependent pathways. In this context, research has indicated that the RAGE inhibitor tetrahydroberberine can reverse cardiac aging by enhancing PHB2-mediated mitophagy and prevents peritoneal adhesions by suppressing inflammation [49]. This evidence for the differing roles of PHB2 in different biological contexts highlights the functional complexity of this protein in cellular processes. In the present study, however, we conclude that overexpression of PHB2 inhibits fibrosis of ARPE-19 cells induced by TGF-β2 by suppressing the AGE–RAGE pathway.

However, although our findings in this study provide valuable insights regarding potential biomarkers for subretinal fibrosis, the study does have certain limitations that should be taken into consideration when interpreting the results. Notably, among the primary limitations is the lack of experimental data regarding the inhibition of PHB2. Owing to constraints associated with sample size and experimental duration, we were unable to conduct knockout experiments for PHB2, which would have enabled us to perform a more direct assessment of the role of this protein in subretinal fibrosis. This limitation may accordingly influence the robustness of our conclusions with respect to the specific functions and mechanisms of PHB2 in the context of subretinal fibrosis. We acknowledge this limitation and are committed to addressing this shortcoming in our future research. Despite this limitation, our findings in this study provide important preliminary data that will lay the groundwork for future investigations into the role of PHB2 in subretinal fibrosis.

## 5 Conclusion

Collectively, our findings in this study indicate that PHB2 would be a promising therapeutic target for the treatment of subretinal fibrosis. Overexpression of PHB2 was found to inhibit subretinal fibrosis by inhibiting the Smad- and non-Smad-dependent pathways in TGF-β2-induced ARPE-19 cells via suppression of the AGE–RAGE pathway. These findings accordingly provide valuable insights into the mechanisms whereby PHB2 alleviates subretinal fibrosis and highlight its potential value as a target for the treatment of subretinal fibrosis.

## Supplementary Material

Supplementary Figure
