# Supplementary material for: PHB2 alleviates retinal pigment epithelium cell fibrosis by suppressing the AGE–RAGE pathway
Source: Open Life Sci. 2024 Nov 4;19(1):20220985. doi: 10.1515/biol-2022-0985 (PMC11538926; doi:10.1515/biol-2022-0985)
Supplement: Supplementary Figure [file biol-2022-0985-sm.pdf]

Supplementary material

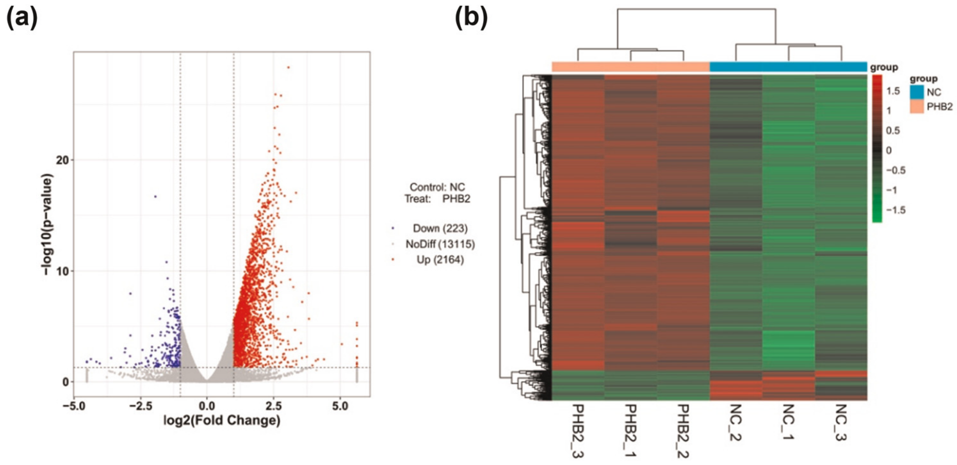

Figure S1: Transcriptome sequencing analysis. (a) Volcano map of differentially expressed genes. (b) Cluster map of differentially expressed genes.

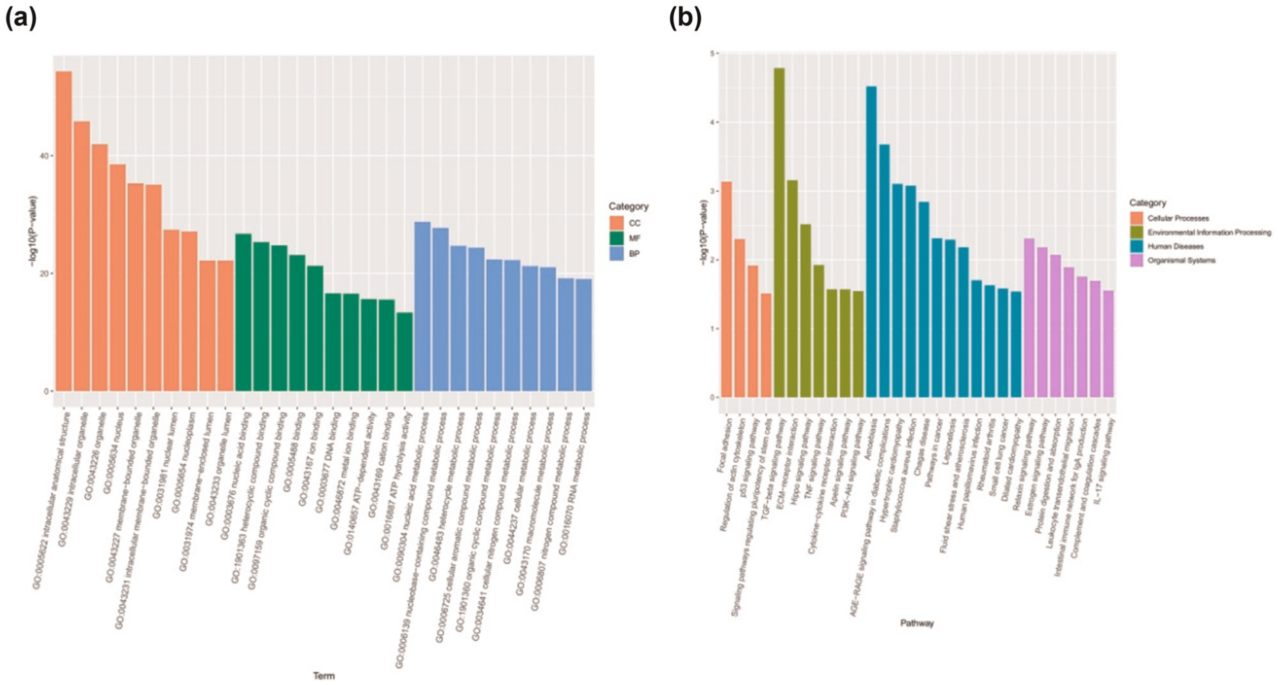

Figure S2: Gene Ontology and Kyoto Encyclopedia of Genes and Genomes (KEGG) enrichment analysis. (a) GO enrichment results. (b) KEGG pathway enrichment results.
